# Catastrophic health expenditure on chronic non-communicable diseases among elder population: A cross-sectional study from a sub-metropolitan city of Eastern Nepal

**DOI:** 10.1371/journal.pone.0279212

**Published:** 2022-12-13

**Authors:** Sangita Rai, Swotantra Gautam, Gopal Kumar Yadav, Surya Raj Niraula, Suman Bahadur Singh, Rajan Rai, Sagar Poudel, Ram Bilakshan Sah

**Affiliations:** 1 School of Public Health and Community Medicine, B. P. Koirala Institute of Health Sciences, Dharan, Nepal; 2 Ministry of Health, Biratnagar, Nepal; 3 Department of Internal Medicine, Advent Health, Orlando, Florida, United States of America; 4 Department of Internal Medicine, B. P. Koirala Institute of Health Sciences, Dharan, Nepal; 5 Ministry of Health and Population, Amphipath, Kathmandu, Nepal; 6 Department of Anaesthesiology and Critical Care, Koshi Hospital, Biratnagar, Nepal; 7 Department of Community Medicine, All India Institute of Medical Sciences, New Delhi, India; IGMC: Indira Gandhi Medical College, INDIA

## Abstract

**Introduction:**

This study was conducted with the objective to analyze the out-of-pocket (OOP) healthcare expenditure and catastrophic healthcare expenditure (CHE) on chronic non-communicable diseases (CNCD) among the elderly population, and the association of CHE on CNCD with associated factors among the same population.

**Materials and methods:**

We collected data from the elderly population of Dharan Sub-metropolitan city of the Eastern Nepal via door-to-door survey and face-to-face interview. The ten wards out of twenty were chosen by lottery method, and the equal proportion out of 280 samples was purposively chosen from each of ten wards (28 participants from each selected ward). The data were entered in Microsoft Excel 2019 v16.0 and statistical analysis was performed by using statistical package for social sciences, IBM SPSS® v21. The chi-square test was used to test the group differences. Multivariable logistic regression was used to determine independent factors associated with CHE (all variables with P < 0.20), and adjusted odds ratios (AOR) were calculated at 95% confidence interval (CI).

**Results:**

The median household, food and health expenditures were 95325 (72112.50–126262.50), 45000 (33000–60000) and 2100 (885.00–6107.50) NPR respectively. The proportion of the participants with CHE was 14.6%. The single living participants had 3.4 times higher odds of catastrophic health expenditure (AOR = 3.4, 95% CI = 1.2–9.6, P-value = 0.022) than those who are married. Similarly, those who had cancer had 0.1 times lower odds of CHE (AOR = 0.1, 95% CI = 0.0–0.2, P-value = <0.001) than those without cancer.

**Conclusion:**

The elder population had significant financial health shocks due to chronic health ailments. There should be the provision of mandatory health insurance programmes for elderly to cut down the catastrophic healthcare expenditure. Similarly, there should be the provision of exemption scheme for vulnerable elderly who are more likely to face catastrophic expenditure from all available health facilities.

## Introduction

Chronic non-communicable diseases (CNCD) like hypertension, cardiovascular diseases (stroke and acute coronary syndrome), diabetes mellitus, chronic obstructive pulmonary diseases (COPD), cancer, etc. contribute to the major burden of disability and death worldwide [1,2]. Due to requirement of long-term treatment and care, the expenditure on CNCD is expensive. Out-of-pocket (OOP) healthcare expenditure is one of the means of financing health expenditure around the world, and particularly developing countries where access to financial protection (provided by government) or health insurance is marginal [3].

Globally the population aged 60 years and above was estimated at 11% in 2007 and set to rise to over 20% by 2050 [4]. For the treatment and management of CNCD for a long period of time to restore the health condition of elderly, they have to pay high OOP to the health provider which leads to financial burden to the household [5,6].

Sustainable Development Goal (SDG) indicator focuses on financial protection, and defines OOP healthcare expenditure as that proportion of the population who has large household expenditure on health from total household expenditure or income [7]. It is viewed as catastrophic according to world health organization (WHO) when it is equal or exceeds 40% of a household’s capacity of pay [8].

In country like Nepal, people have to pay in both public and private sectors in all health services. So, a significant proportion of healthcare financing consists of OOP [9]. Nepal healthcare financing system doesn’t protect household from catastrophic health expenditure owing to low-income nation. The available national social security act, a community-based health insurance (CBHI) of Nepal covers only 5% of population in 2018 and the participation is entirely voluntary. There has been alarming rate of commercialization and privatization of health care, increasing the economic burden on general public for health care [10].

This study aimed to assess the OOP and the catastrophic healthcare expenditure (CHE) on CNCD among elder population, and also helped to identify the associated factors for CHE among the elderly population.

## Materials and methods

### Design

We did a community-based cross-sectional study among people aged 60 years and above, residing in selected areas of Dharan Sub-metropolitan city and having chronic non-communicable diseases from 1^st^ September, 2018 to 31^st^ August, 2019.

### Setting

Dharan is a Sub-metropolitan city located in Sunsari district in the Province No. 1 of the Eastern Nepal with an area of 192.61 km^2^. It has a total population of 137,705 with male and female being 64,671 and 73,034 respectively. It has a total of 20 wards, and 34,834 households as per census 2021 [11,12].

### Study population

Our study included all those participants aged 60 years and above either having hospital/clinic reported diagnosis of chronic non-communicable disease (CNCD) and under prescribed medication for previous three or more months or admitted frequently with same CNCD for more than 2 times in the hospital in last three months. Those not willing to consent to participate in the study and/or whose costs of treatment and medication fully paid by pension, or social security act (community-based health insurance of Nepal) were excluded from the study.

### Sample size calculation

Assuming the prevalence of catastrophic healthcare expenditure (CHE) to be 13.8% [13] with significance level set at 5%, absolute precision of 4% and a finite population correction, the sample size was calculated to be 280. To collect the information from 280 participants, we selected 10 wards out of total 20 wards of Dharan Sub-metropolitan city through lottery method viz., 1, 2, 4, 6, 7, 9, 10, 11, 16, and 19 numbered wards. Equal number of participants (28 participants from 28 different households of each selected wards) meeting the inclusion criteria were approached by door-to-door survey and face-to-face interview method. If there was no elderly with CNCD in the selected household and/or did not give consent, the next household with elderly with CNCD was selected until the sample size was met for that respective ward. If more than one participant were available in the same household, then one participant was selected using lottery method.

### Variables studied

**Dependent variables** were the presence or absence of catastrophic health expenditure. **Independent variables** were **socio-demographic characteristics** which included age, gender, educational status and occupation; **household characteristics** which included religion, ethnicity, marital status, type of family, number of family members, foreign employee, number of children, living with/without children, family income, and poverty line; **health utilization behavior and disease characteristics** which included type of health services used [Allopathic, Allopathic with Ayurveda and other (traditional healer and homeopathic)], type of health facilities (private/public/both) and type of CNCD [hypertension, cardiovascular diseases (heart diseases and stroke), diabetic mellitus, chronic obstructive pulmonary disease (COPD), cancer and multi-morbidity].

### Data collection

Door-to-door survey was done for people with age more than or equal to 60 years with CNCD. They were identified by asking their age by birth year, or citizenship cards. CNCD were identified with the OPD card, hospital reports and/or the drugs list. The data was collected through face-to-face interview with the semi-structured questionnaires. If the respondent was not available in the selected household, three follow-up visits were done. Even after third visit if respondent was not available, next household was selected.

Total household expenditure (THoE/EXP) including total food expenditure or subsistence expenditure (TFE/SE) was calculated for 3 months by taking the reference from 1^st^ month, and out-of-pocket (OOP) healthcare expenses (i.e., total health expenditure, THE) was asked for three months before interview to minimize the recall bias. The OOP did not determine the financial risk of the household so we use the catastrophic health expenditure of its OOP health expenditure was equal to or greater than 40% of its capacity to pay. The WHO recommends a cut-off level of 40% of non-food expenditure for catastrophic health expenditure (8)

### Equations used in the measurement of CHE

*ThoE/EXP: total household expenditure for 3 months*

*TFE/SE: total food expenditure for 3 months/ subsistence expenditure*

*OOP/THE: out-of-pocket payment/ total health expenditure*

*CTP: capacity to pay = ThoE-TFE*

*CHE: ≥40% of OOP/CTP*

### Data entry and analysis

Data were entered into Microsoft Excel 2019 v16.0 (Microsoft, WA, USA) and appropriate commands were used for data cleaning. Entered data was analyzed using Statistical Packages for Social Sciences version 21 (IBM SPSS Corporation, Armonk, New York, USA). Data were presented as frequency, percentages, means ± or standard deviations (SD), medians and interquartile range as necessary. The Chi-square test was used to test for group differences. For univariable logistic regression analyses, odds ratios (OR) and 95% confidence interval (CI) were calculated. Multivariable logistic regression was used to determine independent factors associated with CHE, and adjusted odds ratios (AOR) were calculated at 95% confidence interval (CI). All variables with P < 0.20 were retained in the final multivariable model.

### Operational definitions

Alternative medicine: It comprises traditional healer and ayurvedics

Elderly: All those with age ≥ 60 years

Household expenditure: Expenditure of over a period on goods and services without any special expenditure on funerals, festivals or weddings

Multi-morbidity: ≥2 co-morbid illness/CNCD

Poverty status: The income of 1.9 US $ per person per day (in US dollars) was treated to have poverty status. As of January 2020, $1 USD = **₨114.0452~ Rs. 115** [14,15].

### Ethical approval

It was obtained from Institutional Review Committee of B.P. Koirala Institute of Health Sciences (Code No. IRC/1359/018) that approved the methodology used for this article. The purpose of the study and procedures was explained and written informed consent was gained before commencing the interview. The participants were also being informed that their participation was voluntary. They were assured that their responses would be treated in confidence and anonymity through the use of strict coding measures. All information was kept confidential and consent forms was number coded for identification. The elderly capacity to provide the consent was assessed using 10-item questionnaire, University of California Brief Assessment of Capacity to Consent (UBACC) questionnaire by authors involved in data collection [16]. In case the participant lacked the decision-making capacity to understand and consent to participate in the study, the advance directives or surrogate decision makers were approached.

## Results

The mean age of the participants was 69.7 ± 8.3 years. More than half of the participants (144, 51.4%) belonged to age group 60–69 years. The proportion of female participants was 56.4% (158). More than two-thirds of the participants (185, 66.1%) had informal education. The proportion of house makers and self-employed were 46.1% (129) and 34.3% (96) respectively. Majority of the participants were Hindu (189, 67.5%) followed by Kiranti (51, 18.2%). Half of the participants (178. 51.8%) were from Janajati ethnicity **(Table 1A).**

**Table 1:** A: Socio-demographic and household characteristics of the participants (N = 280). B: Socio-demographic and household characteristics of the participants (N = 280).

Characteristics	Categories	Number of Household (N = 280)	Percentage (%)
**Age**	Mean ± SD (Range)	69.73 ± 8.34 (60–99)	
	60–69 years	144	51.43
	70–79 years	96	34.29
	≥80 years	40	14.28
**Gender**	Female	158	56.43
	Male	122	43.57
**Educational status**	Illiterate/Informal	185	66.07
	Up to Primary school	119	6.79
	Up to Secondary school	22	7.86
	Up to +12/high school	30	10.71
	Bachelor	14	5.00
	Master & above	10	3.57
**Occupation**	House maker	129	46.07
	Job (governmental or non-governmental)	16	5.71
	Self-employed	96	34.29
	Retired/Unemployed	39	13.93
**Religion**	Hindu	189	67.50
	Buddhist	28	10.00
	Islam	2	0.71
	Kiranti	51	18.21
	Christian	10	3.57
**Ethnicity**	Brahmin/Chhetri	68	24.29
	Janajati	145	51.78
	Dalit	56	20.00
	Madeshi	4	1.43
	Muslim	1	0.36
	Others (Thakuri, Sanyasi)	6	2.14
**Characteristics**	**Categories**	**Number of Household (N = 280)**	**Percentage (%)**
**Marital status**	Married	201	71.79
	Unmarried	2	0.71
	Separated	3	1.07
	Widowed	74	26.43
**Type of family**	Nuclear	53	18.93
	Joint	220	78.57
	Three generation family	7	2.50
**Family members**	<5	157	56.07
	5–10	119	42.50
	>10	4	1.43
**Foreign employee**	None	175	62.50
	1 person	83	29.64
	2 persons	18	6.43
	≥3 persons	4	1.43
**No of children/off springs**	0	3	1.07
	1–3	130	46.43
	4–6	125	44.64
	≥7	22	7.86
**Living with/without children**	Alone/couple only	37	13.21
	With son/s	184	65.72
	With daughter/s	23	8.21
	All together	36	12.86
**Family income (Rs.)**	Mean± SD (Range)	65260.71± 103809.10 (10000–1000000)	
	≤30000	81	28.93
	30000–60000	122	43.57
	>60000	77	27.50
**Poverty status**	No	224	80.00
	Yes	56	20.00

Majority of the participants (201, 71.7%) were married. The proportion of nuclear and joint family was 18.9% (53) and 78.6% (220) respectively. More than half of the participants (157, 56.0%) had family members less than five. About three-fifths (175, 62.5%) had no foreign employed family members. About 91.1% (255) had children up to six. Majority (243, 86.8%) lived with at least one member. About two-thirds (199, 71.1%) had monthly family income at least 30000 NRS. About one-fifths (6, 20.0%) lived below poverty status. **(Table 1B).**

About four-fifths (228, 81.4%) utilized allopathic only. The proportion of public and private health facilities used was 14.6% (41) and 45.4% (127) respectively. Less than one-thirds (81, 28.9%) had their no regular checkup for CNCD. The most common types of CNCD were hypertension (90, 32.1%), cardiovascular diseases (39, 13.9%) and diabetes mellitus (42, 15.0%). The mean duration of CNCD was 4.6 ± 3.9 years. About two-thirds of the participants (194, 69.3%) had CNCD ranging from 6 months to 5 years. **(Table 2).**

**Table 2:** Health utilization behavior and disease characteristics of the participants (N = 280).

Characteristics	Categories	Frequency	Percentage (%)
**Types of health services used**	Allopathic only	228	81.43
	Allopathic & Ayurveda	23	8.21
	Others (Traditional healer & homeopathic)	27	9.64
**Types of health facilities used**	Public hospital	41	14.64
	Private hospital	127	45.36
	Both	112	40.00
**Regular checkup for CNCD**	No	81	28.93
	Yes	199	71.07
**Types of CNCD**	Hypertension	90	32.14
	Diabetes Mellitus	42	15.00
	Cardiovascular disease	39	13.93
	COPD	33	11.79
	Cancer	8	2.86
	Multi-morbidity	68	24.29
**Duration of CNCD**	Mean± SD (Range)	4.56 ± 3.85 (0.50–20.00)	
	6 months to 1 year	90	32.14
	>1 to 5 years	104	37.14
	> 5 years	86	30.71

The median household, food and health expenditures were 95325 (72112.5–126262.5), 45000 (33000–60000) and 2100 (885.0–6107.5) NRS respectively. **(Table 3).**

**Table 3:** Distribution of the Mean ± Standard Deviation (SD), Median Cost and Interquartile (IQR) of household, food and health expenditure among participants over past three months.

Parameters	Mean ± SD	Median (Min-Max)	Interquartile Range
**THoE/EXP** ^1^	105698.43 ± 49630.38	95325 (26850–363600)	72112.50–126262.50
**TFE/SE** ^2^	50337.86 ± 22950.13	45000 (6000–180000)	33000–60000
**THE/OOP** ^3^	12549.74 ± 38226.34	2100 (10–412500)	885.00–6107.50
**Allopathic medicine**	12337.21 ± 38101.77	1910 (0–412500)	820.00–5825.00
**Alternative medicine**	212.54 ± 1256.24	0 (0–12500)	0–0
**CTP** ^4^	55360.58 ± 34948.71	48450.00 (8400–258600)	34177.50–69307.50

^1^Total Household Expenditure/ Expenditure.

^2^Total Food Expenditure/Subsidence Expenses.

^3^Total Health Expenditure/ Out-of-pocket expenses.

^4^Capacity to pay.

**Fig 1:**
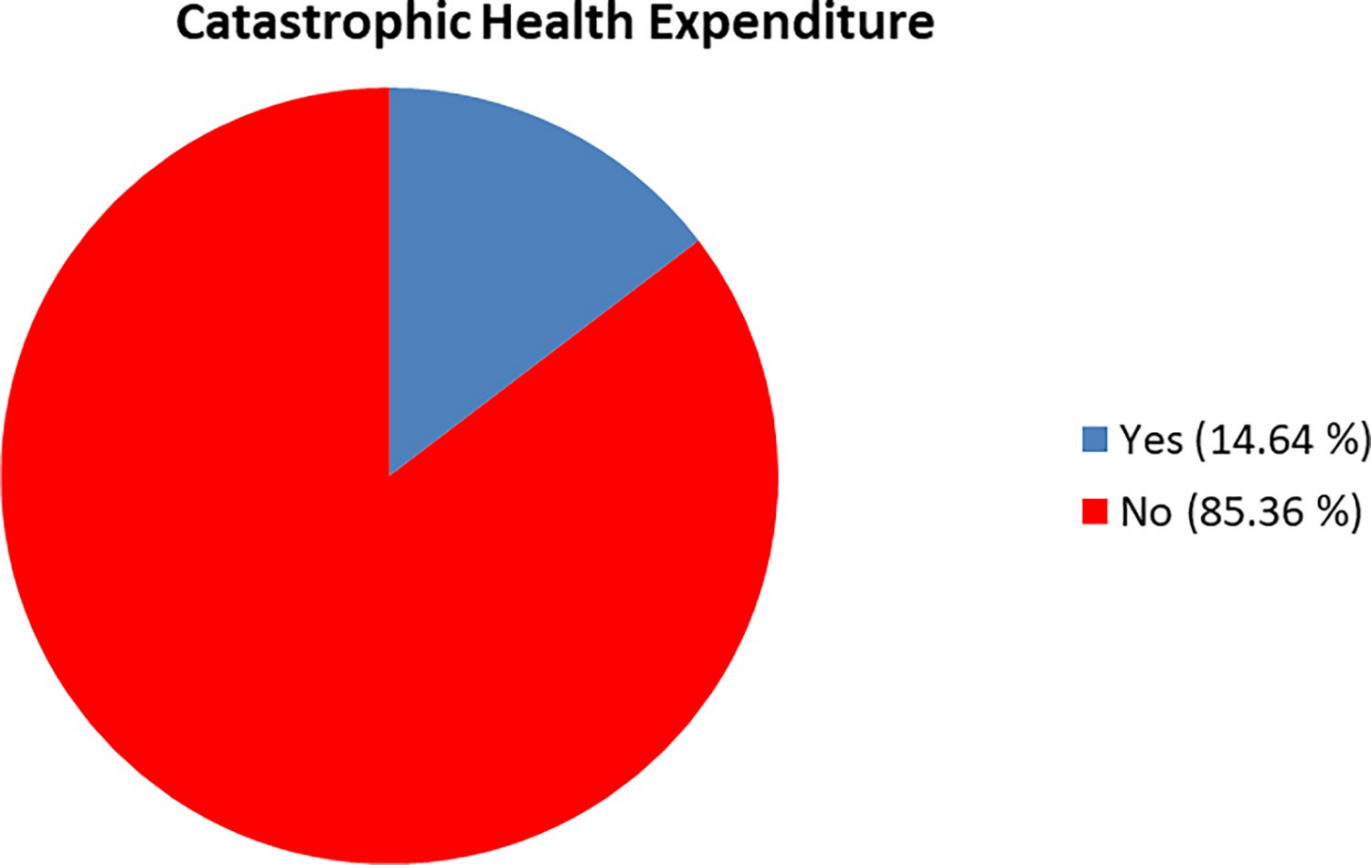
Catastrophic health expenditure of participants.

The proportion of the participants with catastrophic health expenditure was 14.6% **(Fig 1).**

The single participants had 3.4 times higher odds of catastrophic health expenditure (AOR = 3.4, 95% CI = 1.2–9.6, P-value = 0.022) than those who are married. Those who were under poverty status had 0.4 lower odds of CHE (AOR = 0.4, 95% CI = 0.2–0.9, P-value = 0.024) than those who were above poverty line. Similarly, those who had cancer had 0.1 times lower odds of CHE (AOR = 0.1, 95% CI = 0.0–0.2, P-value = <0.001) than those without cancer **(Table 4).**

**Table 4:** Multivariate analysis showing factors associated with out-of-pocket expenditure with or without catastrophic health expenditure (CHE) of chronic non-communicable diseases.

	Multivariable model		
Parameters	AOR	95% CI	P–value
**Marital status**			
Married	1 (Ref.)		
Single^1^	3.38	1.19–9.60	**0.022**
**Poverty status**			
No	1 (Ref.)		
Yes	0.40	0.18–0.89	**0.024**
**Types of health facilities used**			
Public	1 (Ref.)		
Private	0.52	0.16–1.65	0.263
Both	1.06	0.31–3.70	0.922
**HTN**			
No	1 (Ref.)		
Yes	1.38	0.58–3.25	0.466
**CVD**			
No	1 (Ref.)		
Yes	2.28	0.63–8.27	0.212
**Cancer**			
No	1 (Ref.)		
Yes	0.03	0.01–0.22	**<0.001**

^**1**^ **Single: Unmarried + widowed + separated.**

## Discussion

In our study, the mean OOP healthcare expenditure for CNCD was NPR 12549.7 with a standard deviation of NPR 38226.3, and the median and inter-quartile range was NPR 2100 and NPR 5222.5 respectively. Looking at the trends of healthcare expenditure between 1995–1996 and 2010–2011, Nepal’s out of pocket health expenditure increased seven folds, and stands at NPR 3278 [17]. It shows that there is a need to address the non-communicable disease OOP expenditure to protect the household from impoverishment. Our study has comparable finding from one study done in India. The median OOP expenditure from a Haryana state of India was INR 8000, which would be equivalent to NPR 12800 (assuming the average current exchange rate of 1 INR equals to 1.6 NPR) [18]. But another community-based study from West Bengal reported the median OOP health expenditure to be INR 3870. This lower expenditure in West Bengal might be because of taking into consideration the participants with health insurance [19].

In our study about 15% of elderly faced catastrophic health expenditure from OOP expenditure which was the higher than the Nepal living standard survey 2010–2011 which showed 13% of all household suffered CHE [20]. The CHE of rural elderly households with chronic disease patients was higher (30.6%) than the CHE of urban elderly households with chronic disease patients (22.0%) from a study done in China [21]. A study done in India showed that 15.8% of the elderly household incurred catastrophic health expenditure [22]. Based on the repeated cross-sectional analysis of national survey data of India, the OOP expenditure was catastrophic for 17.9% [23].

After adjusting for confounders, we found out that marital status of elderly, poverty status and cancer co-morbid state had significant association with CHE. The single participants had 3.4 times higher odds of CHE (AOR = 3.4, 95% CI = 1.2–9.6, P-value = 0.022) than those who are married. This is comparable to a study from India but with lower strength, where single living elderly had 1.1 times higher odds of CHE than married one [24]. This might be due to the fact that single living elderly did not have either family members or any foreign employed family members to support financially for healthcare. There is an evidence from one study done in China that says the large family size protects against CHE [21].

Our study showed that 20% of elder participants were below poverty status. This is comparable to a study report on “Nepal Multidimensional Poverty Index 2021” that shows 17.4% of Nepal’s population are multi-dimensionally poor. However, such participants under the poverty line had 0.4 lower odds of CHE (AOR = 0.4, 95% CI = 0.2–0.9, P-value = 0.024) than those who were above poverty line. One possible reason for this explanation could be that they already lack budget to spend on their healthcare maintenance. Similarly, those who had cancer had significantly lower odds of CHE (AOR = 0.1, 95% CI = 0.0–0.2, P-value = <0.001) than those without cancer. The explanation for these elderly with cancer is also same. They are already financially deprived to spend on their health ailments. But what might be the actual cause of low CHE in participants with cancer, needs a prospective study to propose a definite hypothesis.

In univariable analysis, the other co-morbid conditions like hypertension, diabetes and cardiovascular diseases posed higher odds of CHE in our study but these data were not statistically significant. Meanwhile, a study from Nepal depicted that in households belonging to the poorest quintile, one or more episodes of diabetes (rate ratio, RR: 2.4; 95% CI: 1.2–4.8), asthma (RR: 2.1; 95% CI: 1.3–3.4) or heart disease (RR: 2.2; 95% CI: 1.3–3.9) were associated with a significantly increased risk of catastrophic expenditure [13]. Similarly, the studies from 15 European countries indicated that diagnosed diabetes mellitus and cardiovascular diseases were significant predictors for catastrophic health expenditure among older people. But cancer was not found to be a significant predictor for catastrophic health expenditure [25]. In contrast, Australian elder who had cancer and diabetic were more likely to face a financial burden than other diseases like hypertension and depression [26].

The most important strength of our study was the most vulnerable participant of the household i.e., the elderly with CNCD who are not entitled to insurance coverage. We had taken the recommended WHO cut-off of 40% of OOP healthcare expenditure to capacity to pay, to determine the CHE. Similarly, this is the first study of its kind to assess the financial shocks in elderly with CNCD from the Eastern Nepal.

Our study has certain limitations. We were not able to assess the changes in financial circumstances over time or the extent to which the financial burdens are rising or falling with changing health conditions owing to the nature of study design i.e., cross-sectional study. There could be more financial burden to the household than revealed in the study as this study was limited to only elder people of the household. Similarly, the household income, expenditure and OOP healthcare expenditure were self-reported, and were not verifiable from another source. So, the reporting bias might had occurred. Additionally, the probability proportional to size (PPS) sampling technique with design effect would have been better adopted to avoid selection bias.

## Conclusions

Elder populations have significant financial risks due to chronic health ailments compared to the general population which calls for financial risk protection mechanisms. Implementation of awareness programme, and preventive strategies for chronic non-communicable diseases could prevent catastrophic health expenditure. There should be the provision for mandatory social health insurance programme for elderly to avoid out-of-pocket healthcare expenses. Similarly, the development of exemption scheme for vulnerable elderly who are more likely to face catastrophic expenditure from all available health facilities could help to reduce health related financial shock.

## Supporting information

S1 Table (DOCX)

S1 Dataset (XLSX)

S1 Questionnaires (DOCX)

## Abbreviations

CHE: catastrophic health expenditure
CNCD: chronic non-communicable diseases
CTP: capacity to pay
NPR: Nepalese Rupee
OOP: out-of-pocket payment
