# Supplementary material for: Catastrophic health expenditure on chronic non-communicable diseases among elder population: A cross-sectional study from a sub-metropolitan city of Eastern Nepal
Source: PLoS One. 2022 Dec 13;17(12):e0279212. doi: 10.1371/journal.pone.0279212 (PMC9747046; doi:10.1371/journal.pone.0279212)
Supplement: S1 Table — (DOCX) [file pone.0279212.s001.docx]

**Table S1A: Association of out of pocket expenditure with or without catastrophic health expenditure (CHE) with socio-demographic and household characteristics of the participants**

|  | **CHE** | | **Univariable model** | | |
| --- | --- | --- | --- | --- | --- |
| **Parameters** | **Yes (%)** | **No (%)** | **OR** | **95% CI** | **P-value** |
| **Age (years)** |  |  |  |  |  |
| 60-69 | 21 (14.58) | 123 (85.42) | 1 (Ref.) |  |  |
| 70-79 | 13 (13.54 | 83 (86.46) | 1.09 | 0.52-2.29 | 0.821 |
| ≥80 | 7 (17.50) | 33 (82.50) | 0.81 | 0.32-2.06 | 0.650 |
| **Gender** |  |  |  |  |  |
| Female | 20 (12.65) | 138 (87.34) | 1 (Ref.) |  |  |
| Male | 21 (17.21) | 101 (82.79) | 0.69 | 0.36-1.35 | 0.287 |
| **Educational status** |  |  |  |  |  |
| Illiterate/informal | 26 (14.05) | 159 (85.95) | 1 (Ref.) |  |  |
| Up to class 10/Secondary | 7 (17.07) | 34 (82.93) | 0.79 | 0.32-1.98 | 0.621 |
| Up to +2 & above | 8 (14.81) | 46 (85.19) | 0.94 | 0.40-2.22 | 0.888 |
| **Occupation** |  |  |  |  |  |
| House maker &Unemployed/Retired | 24 (14.29) | 144 (85.71) | 1 (Ref.) |  |  |
| Job & Self-employed | 17 (15.18) | 95 (84.82) | 0.93 | 0.48-1.83 | 0.836 |
| **Religion** |  |  |  |  |  |
| Hindu | 27 (14.29) | 162 (85.71) | 1 (Ref.) |  |  |
| Others | 14 (15.38) | 77 (84.62) | 0.92 | 0.46-1.85 | 0.808 |
| **Ethnicity** |  |  |  |  |  |
| Brahmin/Chhetri | 13 (19.12) | 55 (80.88) | 1 (Ref.) |  |  |
| Janajati | 20 (13.79) | 125 (86.21) | 1.48 | 0.69-3.18 | 0.319 |
| Others | 8 (11.94) | 59 (88.06) | 1.74 | 0.67-4.52 | 0.254 |

**Table S1B: Association of out of pocket expenditure with or without catastrophic health expenditure (CHE) with socio-demographic and household characteristics of the participants**

|  | **CHE** | | **Univariable model** | | |
| --- | --- | --- | --- | --- | --- |
| **Parameters** | **Yes (%)** | **No (%)** | **OR** | **95% CI** | **P-value** |
| **Marital status** |  |  |  |  |  |
| Married | 36 (17.91) | 165 (82.09) | 1 (Ref.) |  |  |
| Single^1^ | 5 (6.33) | 74 (93.67) | 3.23 | 1.22-8.56 | **0.014** |
| **Type of family** |  |  |  |  |  |
| Nuclear | 8 (15.09) | 45 (84.91) | 1 (Ref.) |  |  |
| Joint & three generation | 33 (14.54) | 194 (85.46) | 1.05 | 0.45-2.42 | 0.918 |
| **Family members** |  |  |  |  |  |
| <5 | 22 (14.01) | 135 (85.99) | 1 (Ref.) |  |  |
| ≥5 | 19 (15.45) | 104 (84.55) | 0.89 | 0.46-1.73 | 0.736 |
| **Foreign employee** |  |  |  |  |  |
| No | 22 (12.57) | 153 (87.43) | 1 (Ref.) |  |  |
| Yes (at least one) | 19 (18.10) | 86 (81.90) | 0.65 | 0.33-1.27 | 0.206 |
| **Living with/without children** |  |  |  |  |  |
| Alone/couple only | 7 (18.92) | 30 (81.08) | 1 (Ref.) |  |  |
| ≥1 member | 34 (13.99) | 209 (86.01) | 1.43 | 0.58-3.52 | 0.430 |
| **Poverty line** |  |  |  |  |  |
| No | 28 (12.50) | 196 (87.50) | 1 (Ref.) |  |  |
| Yes | 13 (23.21) | 43 (76.79) | 0.47 | 0.23-0.99 | **0.043** |

**^1^Single: Unmarried, separated & widowed**

**Table S2: Association of out of pocket expenditure with or without catastrophic health expenditure (CHE) with health utilization behavior, and disease characteristics of the participants**

|  | **CHE** | | **Univariable model** | | |
| --- | --- | --- | --- | --- | --- |
| **Parameters** | **Yes (%)** | **No (%)** | **OR** | **95% CI** | **P-value** |
| **Types of health services used** |  |  |  |  |  |
| Allopathic only | 35 (15.35) | 193 (84.65) | 1 (Ref.) |  |  |
| Others | 6 (11.54) | 46 (88.46) | 0.72 | 0.29-1.81 | 0.484 |
| **Type of health facilities** |  |  |  |  |  |
| Public | 4 (9.76) | 37 (90.24) | 1 (Ref.) |  |  |
| Private | 24 (18.90) | 103 (81.10) | 0.46 | 0.15-1.43 | **0.180** |
| Both | 13 (11.61) | 99 (88.39) | 0.82 | 0.25-2.69 | 0.747 |
| **Regular checkup for CNCD** |  |  |  |  |  |
| No | 15 (18.52) | 66 (81.48) | 1 (Ref.) |  |  |
| Yes | 26 (13.07) | 173 (86.93) | 1.51 | 0.75-3.03 | 0.242 |
| **Types of CNCD** |  |  |  |  |  |
| **HTN** |  |  |  |  |  |
| No | 32 (16.84) | 158 (83.16) | 1 (Ref.) |  |  |
| Yes | 9 (10.00) | 81 (90.00) | 1.82 | 0.83-4.00 | **0.135** |
| **Diabetes** |  |  |  |  |  |
| No | 35 (14.77) | 202 (85.23) | 1 (Ref.) |  |  |
| Yes | 6 (13.95) | 37 (86.05) | 1.07 | 0.42-2.72 | 0.889 |
| **CVD** |  |  |  |  |  |
| No | 38 (15.77) | 203 (84.23) | 1 (Ref.) |  |  |
| Yes | 3 (7.69) | 36 (92.31) | 2.25 | 0.66-7.67 | **0.196** |
| **COPD** |  |  |  |  |  |
| No | 34 (13.77) | 213 (86.23) | 1 (Ref.) |  |  |
| Yes | 7 (21.21) | 26 (78.79) | 0.59 | 0.24-1.47 | 0.260 |
| **Cancer** |  |  |  |  |  |
| No | 35 (12.87) | 237 (87.13) | 1 (Ref.) |  |  |
| Yes | 6 (75.00) | 2 (25.00) | 0.05 | 0.01-0.25 | **<0.001** |
| **Multi-morbidity** |  |  |  |  |  |
| No | 31 (14.62) | 181 (85.38) | 1 (Ref.) |  |  |
| Yes | 10 (14.71) | 58 (85.29) | 0.99 | 0.46-2.15 | 0.987 |
| **Duration of CNCD** |  |  |  |  |  |
| 6 month to 1 year | 13 (14.44) | 77 (85.56) | 1 (Ref.) |  |  |
| >1 to 5 years | 16 (15.38) | 88 (84.62) | 0.93 | 0.42-2.05 | 0.855 |
| >5 years | 12 (13.95) | 74 (86.05) | 1.04 | 0.45-2.43 | 0.926 |
