# Supplementary material for: Catastrophic health expenditure on chronic non-communicable diseases among elder population: A cross-sectional study from a sub-metropolitan city of Eastern Nepal
Source: PLoS One. 2022 Dec 13;17(12):e0279212. doi: 10.1371/journal.pone.0279212 (PMC9747046; doi:10.1371/journal.pone.0279212)
Supplement: S1 Questionnaires — (DOCX) [file pone.0279212.s003.docx]

## ANNEX 1: QUESTIONNIRE

**Catastrophic health expenditure on chronic non-communicable diseases among elder population: a cross-sectional study from a sub-metropolitan city of eastern Nepal**

Name of interviewer:

Municipality:

Ward number:

Household number:

Name of elder person:

Age:

Contact number:

**PART 1: SCREENING QUESTION**

| **SN.** | **QUESTION** | **RESPONSE** | **SKIP** |
| --- | --- | --- | --- |
| 1.1. | What is your full name? |  |  |
| 1.2. | Have you had any chronic non communicable disease? | 1.Yes  2 No | Stop Interview |

**PART 2: HOUSEHOLD CHARACTERISTICS**

**2.1. BACKGROUND INFORMATION**

| **S.N.** | **QUESTION** | **RESPONSE** |
| --- | --- | --- |
| 2.1.1. | What is your family type? | 1.Nuclear Family  2.Joint Family  3.Three generation Family |
| 2.1.2. | What is the total number of members in your family? | 1.less than 5 members  2. 5-10 members  3.more than 10 members |
| 2.1.2 | How many children do you have | - - - 1. 0 children       2. 1-3 children       3. 4-6 children       4. 7-9 children |
| 2.1.3. | What is your total Family income in a month? (including income from agriculture/animal farming or selling of any homemade product) |  |

**2.2. HOUSEHOLD ROASTER**

|  | **Sex** | **Age** | **Education** | **Religion** | **Ethnicity** | **Marital status** | **Occupation** |
| --- | --- | --- | --- | --- | --- | --- | --- |
| Names of elder | 1= Male  2= Female | 1 60- 70  2.71- 80  Above 80 | *** * REFER TO CODE BELOW** | 1=Hinduism 2=Buddhism  3=Islam  4=Kirat  5=Christianity  6=Other | 1 Brahaman/ Chettri  2 Terai / Madhesi  3 Dalits  4. janajati  5. Muslim  6. Others | 1= single  2=married  3=Divorced  4=Separate  5=Widowed | *** * * REFER TO CODE BELOW** |

| ****What is the highest Class in education that (NAME) completed at current age?** |
| --- |
| 1=Illiterate 2=Informal education 3=Primary education 4=Secondary education 5=Higher secondary education 6=Bachelor/Above bachelor |
| *****What is NAME`s main occupation?** |
| 1=Household work 2=Government job 3=Private 4= Retired 5= others |

**PART 3: HOUSEHOLD EXPENDITURE INFORMATION**

| **S.N.** | **QUESTIONS** | | **RESPONSE** | **SKIP** |
| --- | --- | --- | --- | --- |
| Household expenditure for past 30 days (refer to a normal one month period without any special expenditure for festivals, weddings or funerals)  **WRITE IN RUPEES** | | | |  |
| **3.1.** | | **Food Expenditure** | **Past 30 days** |  |
| 3.1.1. | | How much money did your household spend on food bought from outside? (E.g. rice, beans, cooking oil. vegetable, meat etc.) |  |  |
| 3.1.2. | | Did your household consume food that was grown or produced by your household?  1.Yes  2.No  If Yes, how much would your household have spent in the market to buy this quantity of food? |  | Skip to 3.1.3. |

| 3.1.3. | | Did your household consume any food that was received in-kind (gift, donation or wages for work, etc.)?  1.Yes  2.No  If Yes, What is the total value of food consumed that your household received in-kind (gift, donation or wages for work, etc.)? |  | Skip to 3.2. |
| --- | --- | --- | --- | --- |
| **TOTAL FOOD EXPENDITURE** | | |  | |
| **3.2.** | | **How much money did your household spend on:** |  |  |
| 3.2.1. | | Education |  |  |
| 3.2.2. | | Clothes and foot wear |  |  |
| 3.2.3. | | Personal care items (soap, shampoo, toothpaste, cosmetics, haircuts and the like) |  |  |
| 3.2.4. | | Household items (laundry soap, cleaning items, anti-mosquitoes and the like) |  |  |
| 3.2.5. | | Water, Electricity , fuel and sewage |  |  |
| 3.2.6. | | Transportation |  |  |
| 3.2.7. | | Telecommunication fee |  |  |
| 3.2.8 | | Rent |  |  |
| 3.2.9 | | Health care and treatment costs |  |  |
| 3.2.10 | | Socializing and recreation (except funerals, dowries and weddings) |  |  |
| 3.2.11 | | Loan repayment |  |  |
| 3.2.12 | | Others (specify) |  |  |
| **TOTAL EXPENDITURE (B)** | | |  |  |
| **3.3.** | **Did your household received any amount in-kind (gift, donation or wages for work, etc.) for any of the above as mentioned in 3.2?**  01.Yes  02.No  If Yes, What is the money value of the amount received in-kind (gift, donation or wages for work, etc.) by your household for: | |  | Skip to Part 3.4 |
| **TOTAL EXPENDITURE (C)** | | |  | |
| **3.4.** | **TOTAL HOUSEHOLD EXPENDITURE (A+B+C)**  **[ IN A MONTH ]** | |  | |
| **3.5.** | **TOTAL FOOD EXPENDITURE IN MONTH (A) * 3**  **[ IN 3 MONTH]** | |  | |
| **3.6.** | **TOTAL HOUSEHOLD EXPENDITURE (A+B+C) * 3**  **[ IN 3 MONTH]** | |  | |

**PART 4: RESPONDENT EPISODES OF ILLNESSES (PAST 3 MONTHS)**

| **INFORMATION OF ILL MEMBERS OF RESPONDENT DURING PAST 3 MONTHS** | |  |
| --- | --- | --- |
| **DIAGNOSIS (IF AVAILABLE) OR MAIN SYMPTOM** | **DURATION OF ILLNESS** | |
|  | **MONTH** | **DAY** |
|  |  |  |

**PART 5: HEALTH PROBLEMS AND HEALTH UTILIZATION BEHAVIOUR (PAST 3 MONTHS)**

| **5.1.Diagnosis** | **5.2. Did (NAME) visit somewhere to seek care?**  1=Yes  2= No (Skip to 5.8) | **5.3. Where did (NAME) seek care for this illness in the past 3 months?**  1=Allopathic  2= Homeopathy  3=Ayurveda  4=Traditional Healer  5= Home remedy  (Multiple Response) | **5.4. Where did NAME seek care for this illness in the past 3 months? (for allopathic only )**  01= Private Health Facilities (Specify)  02= Public Health Facilities (Specify)  3=Both Private and Public | **5.5.Why did (NAME) not go to seek care?**  *** REFER TO CODE BELOW  (Multiple Response) |
| --- | --- | --- | --- | --- |

**PART 6: HEALTH EXPENDITURES (PAST 3 MONTHS ALL CNCD)**

**6.1. HEALTH EXPENDITURES (ALLOPATHY)**

| **Episode of illness** | **Was there any allopathic cost incurred due to the illness in the past 3 month?**  01=Yes  02=No (Skip to 6.2) | **How much did the household pay for treatment of the illness in the past 3 months (Include all costs for modern medicine)? Please do not include hospitalization which is defined as having spent at least one night in hospital**. **RECORD IN RUPEES. IF NOTHING WAS SPENT WRITE 0.** | | | | | | **Total heath expenditure (allopathy)**  **A= a + b** |
| --- | --- | --- | --- | --- | --- | --- | --- | --- |
|  |  | **Direct cost** | | | | **Total Direct COST (A)** | **Indirect cost (b)** |  |
|  |  | Fees including consultation/ investigation fee, diagnosis & test (e.g. X ray, blood test RBS etc) | Cost of drugs and medical supply | Transport cost for (NAME) and accompanying family members | Other costs (specify) |  | Wage lost of any member of lness |  |
|  |  |  |  |  |  |  |  |  |
|  |  |  |  |  |  |  |  |  |
|  |  |  |  |  |  |  |  |  |

**6.2. HEALTH EXPENDITURES (ALTERNATIVE MEDCINE)**

| **RECORD IN RUPEES** , **IF NOTHING WAS SPENT WRITE 0** | | | | | **TOTAL HEATH EXPENDITURE (ALTERNATIVE MEDICINE)**  **B= a+ b** |
| --- | --- | --- | --- | --- | --- |
| **Was there any cost incurred due to utilization of alternative medicine (Traditional medicine Healer, Ayurveic) due to the illness in the past 3 months?**  01=Yes  02=No (Skip to 6.3) | **TRADITIONAL MEDICINE AND OTHER HEALER (a)** | | | **AYURVED (b)** |  |
|  | How much did the HH pay in cash for traditional healer for the illness in the past 3 months? | Did the HH pay in kind for traditional healer for the illness?  If so, how much would the household have spent in the market to buy the item? | total | How much did the HH pay for Ayurvedic treatment for the illness in the past 3 months? |  |
|  |  |  |  |  |  |

**6.3. HEALTH EXPENDITURES (INPATIENT)**

| **Did (NAME) receive inpatient treatment for the illness in the past 3 months?**  1=Yes  2=No (Skip to 6.4) | **No. of times hospitalized**  1= 1 time  2= 2 times  3= 3 times  4= more than 4 times | **No. of days spend in the hospital**  **1 (1-2)**  **2(3-4)**  **3(4-5)**  **4(6-7)**  **More than 7days** | **How much did the household pay for hospitalization due to this illness in the past 3 months? Hospitalization refers to having spent at least one night in hospital.**  **RECORD IN RUPEES. IF NOTHING WAS SPENT WRITE 0.** | | | | | | | | | **TOTAL HEALTH EXPENDITURE (INPATIENT)**  **C= a + b** |
| --- | --- | --- | --- | --- | --- | --- | --- | --- | --- | --- | --- | --- |
|  |  |  | **DIRECT COST** | | | | | | | **TOTAL DIRECT COST (a)** | **INDIRECT COST(b)** |  |
|  |  |  | **Medical cost** | | **Living cost for NAME during hospitalization** | | **Expenses for accompanying** | | **Other charge**  **(specify)** |  |  |  |
|  |  |  | Fees including consultation/ investigation fee , bed fee, diagnosis & test | Cost of drugs and medicine | Food | Others (Specify) | Food & lodging | Transport  (including transport cost for NAME) |  |  | Wage lost of any member of HH due to illness |  |
|  |  |  |  |  |  |  |  |  |  |  |  |  |
|  |  |  |  |  |  |  |  |  |  |  |  |  |
|  |  |  |  |  |  |  |  |  |  |  |  |  |

**6.4 Out-of-pocket payment**

**PART 7: CALCULATION OF CATASTROPHIC HEALTH EXPENDITURE (PAST 3 MONTHS)**

| **Household consumption expenditure** | | **Household capacity to pay (CTP)** | **Health expenditure** | **Household catastropic health expenditure (CHE)** |
| --- | --- | --- | --- | --- |
| Total food expenditure of HH in past 3 month (subsistence expenditure: SE)  **Refer to q.3.5** | Total household consumption expenditure in past 3 months (exp)  **Refer to q.3.6** | CTP= EXP- SE | Total health expenditure in past 3 month  (out of pocket payment :OOP)  **Refer to q.6.4** | CHE occurs if  OOP/CTP > 40%  01= presence of household CHE  02= absence of household CHE |
|  |  |  |  |  |
